# Doctoral research, COVID-19, and political crisis in Ethiopia, Sudan, Rwanda, and the UK: challenges, responses, and recommendations

**DOI:** 10.3310/nihropenres.13470.1

**Published:** 2023-10-11

**Authors:** Jean Paul Bikorimana, Corinna Thellmann, Tseganesh Mulugeta, Dereje Wonde, Addisu Tsegaye, Badraldeen Ali Bashir Alnoor Ahmed, Ursin Bayisenge, Jeffrey Pocock

**Affiliations:** 1 Social Sciences for Severe Stigmatising Skin Diseases (5S) Foundation, Center for Health Research, Brighton and Sussex Medical School, University of Sussex, Brighton, BN1 9PX, UK; 2 College of Social Sciences, Addis Ababa University, Addis Ababa, Ethiopia; 3 ThinkWrite, 18 Rosewood Ave, Alveston, Bristol, BS35 3PP, UK

**Keywords:** Collaborative autoethnography. Lived experience. Doctoral research. COVID-19. Ethiopia. Rwanda. Sudan. Global South

## Abstract

**Background:**

Conducting doctoral research is a challenging endeavour, a challenge which as the growing literature on the subject has shown, the COVD-19 pandemic has made even more so. For some doctoral researchers, however, the pandemic has also been accompanied by political unrest and military conflict, putting them and their networks at risk and making their research especially difficult to sustain.

**Methods:**

We have used a collaborative auto-ethnography, and we, a group of seven doctoral researchers based in Ethiopia, Rwanda, Sudan and UK have written our experiences.

**Results:**

Drawing upon the results of a collaborative auto-ethnography (CAE), this article records and discusses the experiences of a group of doctoral researchers who with the support of their organisation, the Social Science for Severe Stigmatised Skin Diseases (5S) Foundation, have been attempting to cope with both the pandemic and internal instability and strife. After firstly setting the context, the article explains why for the purposes of this paper CAE was adopted as our method, and then documents and discusses the experiences of seven doctoral researchers based in Ethiopia, Sudan, Rwanda, and the UK, doing so in terms of four different themes: New Ways of Working and Its Impact, Change and Delay, Mental Health and Well-Being Impact, and Qualities and Capacities.

**Conclusion:**

What these experiences tell us is that this group of doctoral researchers have found themselves in extremely challenging situations, which have placed exceptionally high demands on them and their support networks, and this has had an impact on their health and well-being although also been the catalyst for some more positive development. Given their lived experiences, the article finishes with a series of recommendations for future research projects of this kind.

## Introduction

Conducting doctoral research is no easy task. The PhD journey has been described as a “marathon process” that involves tiredness and stamina within the framework of time restrictions and demands of academic work and life ( Glaze, 2002). It is perhaps unsurprising then that doctoral students often report feelings of loneliness and social isolation ( Litalien & Guay, 2015; McAlpine & Amundsen, 2009), they struggle to balance the demands of their PhDs and their other relationships and responsibilities ( Brus, 2006; Rizzolo *et al*., 2016), and are prone to stress and anxiety ( Sverdlik *et al*, 2020).

It is perhaps also unsurprising that these longstanding issues have been aggravated by COVID-19. Among other things, through the course of the pandemic students have reported difficulties in continuing their research ( Byrom, 2020; Cahusac de Caux, 2021; Eigege & Kennedy, 2021), especially for those conducting fieldwork or in-person research ( Levine *et al*., 2021), and reported issues with their mental health ( Byrom, 2020; Chirikov *et al*., 2020; Li *et al*., 2021).

In higher education, one challenge has been how to continue doctoral programmes under the various restrictions on groups and in-person activities countries have imposed. Universities have responded to varying degrees of success by shifting training and supervision online ( Saberi, 2020). Indeed, the advent of the pandemic has boosted the legitimacy and reputation of online education and provided major impetus to a digital future for tertiary education ( Ewing, 2021). The increasing digitisation of higher education has been especially convenient and efficient for students in developed countries, where the required infrastructure is on hand ( Aristovnik, 2020).

Despite a range of positive outcomes of off-campus, virtual education, especially for Global North students, the benefits of the “new normal” ( Aristovnik, 2020) have not been evenly distributed if reaped at all. The shift to online learning has exacerbated issues of isolation (e.g. Huang *et al*., 2020), motivation (e.g. Korkmaz & Toraman, 2020), and mental health (e.g. depression, anxiety; Odriozola-González *et al*., 2020). Confinement due to COVID-19, for example, has greatly impacted the social, psychological, and physical wellbeing of learners of higher education ( Aristovnik, 2020). According to Masalimova *et al*. (2022), learners studying from home have experienced anxiety, stress, and attention problems. There has also been scepticism regarding the capacity of distance education to allow exchange of feedback, to communicate, to interact, and to attend courses ( Beltekin & Kuyulu, 2020). As well as limited face-to-face-interaction ( Didenko *et al*., 2021), other disadvantages of e-learning include technical challenges ( Adnan & Anwar, 2020) and lack of skills to use available platforms ( Lassoued *et al*., 2020; Mishra *et al*., 2020). It is recognised also that there is inequitable access to the necessary infrastructure, internet and power, which coupled with digital literacy deficits, create ‘incompatibility issues’ ( Mishra *et al*., 2020). Low-income countries’ capacity to exploit online education, in particular, is, among other things, hampered by high Wi-Fi costs, and electricity and equipment shortages ( Hashemi & Kew, 2021).

In addition, the pandemic has affected the conducting of research, leading to temporary suspension or even complete cessation of research activities, especially those involving human participants. The PhD students whose research projects involve extensive fieldwork and interactions with the study participants have grappled with lockdown policies and strict safety precautions put into place to control the pandemic. In such strict conditions, researchers have made amendments to their research design and methods that include shifting from face-to-face interviews to online or phone interviews ( Lin *et al*., 2020).

However, for some doctoral students the impact of COVID-19, the shift to online learning, the closure of campuses, disruption of research plans and preparation, as well as the typical challenges caused by doctoral research have been worsened even further by political crisis and military conflict in their home countries. Conducting doctoral research during times of internal instability like this is a major cause of stress and anxiety regarding the continuation of and effect on the research itself but also regarding the health, well-being, and personal safety of the researcher and their friends, families, and dependants. Crisis and conflict contribute to a fall in enrolment rates and educational budgets, damages educational infrastructure, and has been linked to technological crisis, and low electronic resource usage ( Gul *et al*., 2013). Access to online resources is critically affected by the failure of technology when students cannot visit their academic institutions, and internet blackouts are especially common during periods of internal political strife ( Kessler, 2019).

The article that follows documents the major challenges one group of doctoral students registered in the UK, but based in Ethiopia, Sudan, and Rwanda, as well as in England, faced while attempting to continue their training, supervision, and research at a time of crisis and conflict. More specifically, this article draws on the results of collaborative autoethnographic research carried out on and by seven PhD students - three Ethiopians, one German, two Rwandese and one Sudanese - regarding their experience of doctoral study amid COVID-19, political instabilities and military action. These doctoral students are part of the Social Science for Severe Stigmatised Skin Diseases (5S) Foundation ( Zaman *et al*., 2020) established by the Centre for Global Health Research at Brighton and Sussex Medical School (BSMS), and participating in a UK based university coordinated multi-country global health project drawing on social science methods and face-to-face research. All but one of the participants is based at BSMS. While COVID-19 has been a common problem for all seven students, political instabilities, and military conflict in this research refers specifically to the domestic situations of Ethiopia and Sudan, most recently since 2020 ( African Union, 2022; The United Nations Office for the Coordination of Humanitarian Affairs Services, 2021). The unrest in both countries has had an especially profound impact on the five students whom live in the countries where it has been taking place.

## Methods

### Collaborative autoethnography

We have used a collaborative autoethnography (CAE) approach to document our COVID-19 and political and military unrest-related experiences lived during our PhD training journey. CAE is an extension of the autoethnographic research method. Autoethnography is a study of a self that is grounded in stories/experiences collected from an individual's own life experience ( Hernandez *et al*., 2017). The self-stories/experiences are central to autoethnographers as they rely on self-stories/experiences to shed retrospective light on the ways we are connected to the external world ( Hernandez *et al*., 2017).

A profitable way to unpack the ways in which socio-cultural realities shape researcher stories/experience is to use multiple self-stories/experiences through a collaborative autoethnography (CAE). As the 5S participants are on the same scholarship, CAE offers us the opportunity to reflect on ways our socio-cultural realities shape our perspectives, behaviours and stories. CAE allows researchers to gather self-stories and interpret them in a collective manner ( Hernandez *et al*., 2017) and its use by qualitative researchers has been especially encouraged at unprecedented times like these ( Roy & Uekusa, 2020). In so doing, the CAE creates the spaces for us to strengthen our friendship and illuminate the ways our experiences inform our actions and lives during this critical time. CAE is also especially well-suited to giving voice to the powerless and marginalised. By sharing experiences, it helps a group of people to highlight commonalities in their experiences, identifying where change is required. CAE offers the less powerful a chance to have a more significant, collective say in organisational trajectories, practices and policies and to promote collective action in campaigns for social justice ( Lapadat, 2017). As all contributors are “vulnerable” in sharing experiences, the power differential between them is also levelled, and this in turn fosters the building of a team and of trust ( Lapadat, 2017). Furthermore, as Chang *et al*. (2012) explain, a trusting and supportive team environment can be ‘cathartic’, especially helpful when the research is focused on sensitive or difficult areas.

There are some supposed methodological and ethical limitations involved in the use of autoethnography and CAE, which we recognise, but limitations further that our specific project is perhaps not as susceptible to. The issue of privilege and non-representativeness, for example, applies less in our context as we are almost all Global South researchers conducting our research in our respective countries at a time of global and local crises, and crises which we are in the middle of. In this respect, we give voice to ourselves, the more marginalised and vulnerable of the researcher community, albeit from a position of privilege vis-à-vis our relation to others locally. Ideally, it is suggested ( Chang *et al*., 2012), CAE members should number no more than five so that coordination and collaboration does not become overly complex and too challenging, but as a group of seven we have not encountered any significant problems. In fact, despite our supposed selectivity, subjectivity, “potential for narcissism and self-indulgence”, and unavoidably emotionally-charged readings of our experiences of crisis ( Roy & Uekusa, 2020), we have, among other things, been able to identify in our experiences overlapping themes and intersubjective commonalities.

As is also recommended ( Roy & Uekusa, 2020) we have been measured in our preparation, planning, and practice and so further addressing these methodological and ethical limitations with CAE. There has been “frequent and open communication among the members” ( Roy & Uekusa, 2020), for instance. We have interpreted and scrutinised data collaboratively and employed shared documents to record thoughts and ideas in the same way a research diary might be used. Participation has been completely voluntary, the focus of our collaboration has secured the agreement of all, and has been conducted non-hierarchically and non-coercively ( Lapadat, 2017).

Initially, we recorded our individual stories and reflections independently, with each of us writing down a summary of our experiences. We started planning for our collaborative research and writing our experience in September, 2021, and the manuscript had been fully written in June, 2022. During biweekly meetings we updated each other on our personal experiences, and shared our stories in a group, and shared minutes. This prompted questions and allowed a deeper understanding of the reflected issues in each story. After sharing our stories in the group, all stories and reflections were combined into a shared document text, which we then analysed and thematised.

A standard thematic analysis ( Braun & Clarke, 2006; Braun & Clarke, 2021) has also been carried out both individually and collectively ( Hernandez *et al*., 2017). After the combined document was shared, each read the document and created initial codes. The initial codes were combined and distributed and from this a master list of themes was created. Overarching themes were then identified and shared with our advisors for suggestions and further input.

Similarly, the manuscript has also been completed individually and collectively. Initially, our article was divided into sections, with each of us assigned a section. This helped us save time and ensure collaboration ( Hernandez *et al*., 2017). Later the sections were combined to produce a final shared document text to be reviewed, edited and approved collectively.

## Results and discussion

We have divided our lived experience during this time of instability and upheaval into the following four themes: New Ways of Working and Its Impact, Change and Delay, Mental Health and Well-Being Impact, and Qualities and Capacities.

### 1. New Ways of Working & Its Impact

The COVID-19 pandemic and, in Ethiopia and Sudan, political and military upheaval have severely impacted our PhD research. The upheaval caused by the pandemic, in particular, has forced us to adopt new ways of working, the use of virtual learning technologies and working from home, and new ways not always with positive results. 

***Infrastructure***

The transition to online learning has been especially problematic. One key issue for us has been the greater pressure this has placed on already fragile local internet services and electricity infrastructure. In Ethiopia, for example, it has been revealed previously that online education is a luxury for Ethiopian students, 80% of whom are living in rural areas without access to electricity and essential electronic devices. According to Global Broadband Speed League (2021), Ethiopia, Sudan, and Rwanda, with an average download speed of 1.2 Mbps, 1.8Mbps and 6.28 Mbps respectively, have among the slowest internet speeds on the continent. Already fragile infrastructure coupled with comparatively slow internet connections have in the cases of Ethiopia and Sudan been placed under even greater stress, however, by political and military events.

***Ethiopia***

In Ethiopia, Dereje, for example, has experienced “frequent power and Internet blackouts”, disrupting online meetings. Internet access, he explains, is provided by only one company in the country, the government owned Ethio telecom, and internet demand “skyrocketed” during the lockdown. He writes of having to wait “in a long queue” to gain access to the Wi-Fi modem in his house. The skyrocketing demand and queues for access this has caused is, however, Dereje explains, compounded by the “frequent” power blackouts that disrupt internet connections and meetings and workshops further. Further exacerbating this has been the unstable political situation in the country, which led to shutting the internet down completely at one point. As Dereje writes, in June 2020 the Oromia region and capital city Addis Ababa suffered further political upheaval and one of the government’s responses was to shut down for more than two weeks the internet throughout the country, including university local area networks. In Dereje’s case this internet blackout coincided with the date of submission of his research plan and as a result he was unable to submit it on time.

Unsurprisingly, a number of us, Dereje included, write of how internet access and connection issues have been a cause of significant worry, not least when there are important deadlines approaching, or workshops, training, seminars, or supervisory meetings planned. Indeed, when in the middle of these important meetings, where we may be presenting research progress, for instance, there has been the not unreasonable worry that internet connectivity would be lost.

Tseganesh, for example, tells us that:

I had to present my PhD research proposal online. The worst part is the network. One of the examiners was lost after I started presenting my work. He couldn’t join the session. At that time, I had only one supervisor. Because of an internet blackout, he couldn’t make it as well. I had to finish the defence session with one examiner and the chairperson. If it was face to face, this problem may not be a challenge at all.

The crux of the connectivity issue in Ethiopia, according to Addisu, is attributed to the government’s view of social media as a major force that “fuels violence” in the country. He related this to the situation in western Oromia, where there had been an exclusive interruption of the internet, for the area has long been associated with political instability. Given repeated internet blackouts in the area, where his hometown is also located, Addisu also recalls how he struggled to attend online classes, seminars, trainings and supervisory meetings.

***Sudan***

Based in Sudan, Badraldeen writes of similar issues with internet access and connections caused by much greater pressure on already fragile infrastructure. As in Ethiopia, this has been compounded by political instability and electricity shortages as well as blocking of roads and bridges. He writes that he lost internet access, “when there was a confrontation between the military and the civilians, or the party's leaders announced a demonstration in the capital Khartoum.” In late 2021 he continued to be “extremely affected” by connection to the internet being cut off. At one point, the entire telecommunication network was shut down for more than three weeks. “These setbacks,” he writes, “completely prevented me from attending my session and other activities (workshops and training) with BSMS.”

Because of the precarious nature of Sudan's infrastructure, Badraldeen adds, the rainy season and floods and rainfall that can follow have a greater impact on everyday life. During the last rainy season in 2020, the central capital of Sudan experienced flooding and heavy rainfall, and from June to late September 2020 the intense amount of rain destroyed roads and some residential areas. A large number of people lost their livelihoods and some lost family members. At this time there were also electricity shortages, as the electricity supply was not sufficient for the country. In fact, he explains, such was the lack of electricity supply that local authorities used to switch the electricity supply between different areas in Khartoum and he suffered from a lack of electricity for several hours a day. 

Another issue making matters worse for Badraldeen in Sudan has been rising fuel prices. In response, for electricity and to access the internet Badraldeen would go to the Mycetoma Research Centre (MRC) in Soba, the southern part of Khartoum city, about 14 kilometres from the city centre but 26 kilometres from his house. However, to reach the MRC costs a lot in fuel, as to avoid the blocked roads he had to drive for more than one and half hour to reach the MRC, and fuel costs which he paid out of his own pocket. This has had other consequences, which again have compounded matters, as this impacted his car's performance and it needed spare parts and repairs to keep it running. Queuing for fuel and other basic items also took up time, “several hours”.

While Badraldeen has made use of various technologies to continue his PhD research, Zoom, Telegram, WhatsApp, Messenger, Microsoft Teams and Doodle, using the internet itself also remains very costly. As he explains,

These applications require high speed internet connections, to obtain these types of the internet we have to pay a lot of money to the telecommunication companies. As a student I cannot afford paying such tax

***Rwanda***

If not as unstable politically, in Rwanda, where Ursin and Jean Paul have been based, the infrastructure situation has been similar. In particular, there has been the same frustration and anxiety that the internet would be cut off, or as Jean Paul explains, that the internet connection would be too slow or unstable. So frustrating and worrying has been the experience regarding internet connectivity during the pandemic that, he writes, it has had a “significant effect on me.” So much so that he no longer trusts his home router or cell phone internet connection. “When I thought about an online meeting,” he explains, “I am always worried about the internet.”

He too has had to travel a long distance in order to access the internet for his PhD research, explaining that,

I travelled 2 hour 30 minutes’ drive from my home town Musanze, in north part of Rwanda, to Kigali where the headquarters of the University of Rwanda is located to use the internet facilities at the university for my second scheduled time for presentation.

Jean Paul also recounts how he experienced difficulties accessing resources. Online libraries required some sort of software to download an article and books were available in libraries but not digitalized and so not available online. As result, he writes, the 5S Foundation management purchased some books and shipped them to Rwanda, but there have been restrictions on travel between districts because of COVID-19 and so it was difficult to travel from his home town to Kigali, the capital of Rwanda where the University of Rwanda to which he is affiliated is located. This has meant employing transport agencies to deliver books, as these had been allowed to operate during the inter-districts travel restrictions here in Rwanda.

## Working from home

The infrastructural issues many of us have been facing have compounded issues related to working from home using new technologies. The shift to online training and supervision has led to issues with time zones, for example. The shift to working from home on the one hand allowed us a space with which to continue our studies but has presented a number of problems and not always had positive consequences. Dereje, for example, used to going into the office to work, found the transition to online home working a ‘struggle’. Working at home meant that he could not control his desire to sleep nor maintain previous levels of efficiency and concentration.

Key issues for us working from home during the pandemic have been the availability of space and the layout of our homes. A number of us – Dereje, Jean Paul - have had difficulty ensuring a clear division between work and home life because of shared spaces and children, and has meant creating new spaces elsewhere in the home (Jean Paul). In Tseganesh’s case, her father and mother both required to be strictly isolated given their medical conditions, but this made it very difficult to do her PhD reading and research at home. She considered using the university library, but worried about catching COVID-19 and spreading it at home. She considered renting a home but was experiencing financial issues that made this impossible. She then decided to stay at home permanently and study at night, but found it difficult to sleep during the day with so many people at home.

Working from home because of the pandemic has also placed our relationships with family and friends under strain. Tseganesh, fearful of contracting COVID-19 and spreading it to her parents, could not meet with friends. Jean Paul’s wife had to pretend to her daughter that her father was not in the house in order to do some PhD work. Addisu feels like working from home during the pandemic was “confinement at home” and rather than allowing more time to be with loved ones, was in fact a form of “alienation of oneself, from extended families, friends, and even neighbours.”

In Sudan, Badraldeen writes of this issue in more positive terms, however. He acknowledges that COVID-19 has had a negative impact on his relationships with family, and seeing his father sick with COVID-19 has been difficult, but being at home together has meant a chance to communicate effectively with family members. Indeed, he believes, “it might be for the first time after I left them for schooling that I get to know their hopes, plans, ambitions and how they have gone through various experiences in their lives.”

### 2. Change & Delay

In our stories, there were also commonalities regarding the themes of change and delay. In particular, the pandemic and political and military unrest affected our research sites and conducting of fieldwork and research activities.

***Research sites***

One consequence of the pandemic and political situation was to delay visits to research sites and in one case led to the choice of country being changed altogether. This delay and uncertainty has taken its toll psychologically. This is revealed in the literature, also. Alamgir (2017) underlines how political insecurities in a country, Bangladesh, can be disruptive, with such insecurities creating panic and affecting travel within and outside the study site.

Corinna originally planned to do her fieldwork in Ethiopia. However, the political situation in Ethiopia made things very complicated and eventually she had to change the whole plan of her PhD project. The long-time plan to go to Ethiopia seemed impossible because of the unpredictability of the situation. This, in turn, affected her dream of visiting the country, meeting Ethiopian people and working on her project. Even if Corinna has never been to Ethiopia, she explains, the “unexpected turn of events” made her feel a ‘break-up’ with the country. 

Jean Paul’s story was similar to Corinna’s. With travel restrictions within districts in Rwanda, he was unable to visit his study setting in which he was supposed to go to gather data for his research (“The access to all kinds of aspects was severely affected by the restrictions enforced to contain the COVID-19 pandemic.”)

***Fieldwork and research activities***

In our personal experiences of trying to conduct PhD research at a time of health and political crisis, a further strand to the theme of change and delay related to fieldwork and research activities. Dereje, for example, was unable to contact key informants for his research and other important people who had direct contact with the community, “some of the officials of health bureaus at the regional and zonal level”, as they had “either joined the government forces in the warfront or engaged in community mobilisation activities.” The expansion of conflict in the northern part of Ethiopia forced the government to declare a state of emergency, and this state of emergency also severely affected Dereje’s fieldwork activities, and he was left not knowing when he was going to start his fieldwork. Despite gaining all the required ethical approvals, a process that takes a number of months, he was unable to undertake field activities and eventually postponed his data collection. He had to follow “a wait and see approach to proceed with the fieldwork” until he received updates about the current situation. Addisu also experienced delays to his research activities and fieldwork because of ‘the volatile nature’ of political instability in Ethiopia and the internal conflict in his specific study area.

### 3. Mental Health and Well-Being Impact

All of these changes caused by COVID-19, and political and military strife, to our ways of working and the pressure and strain it has placed us and our relationships under, have unsurprisingly had a big impact on us psychologically. Indeed, diminished levels of wellbeing among doctoral researchers due to the COVID-19 pandemic have been reported in numerous studies ( Atkinson *et al*., 2021; Donohue *et al*., 2021). Other studies have also documented similar psychological challenges among students in the COVID-19 era. For instance, increased overall stress levels ( Guintivano *et al*., 2021), experiences of high levels of mental and physical exhaustion ( Stamp *et al*., 2021), feelings of loneliness and exclusion ( Pyhältöa *et al*., 2022), and anxiety and fear ( Kee, 2021) have all been documented. Poor work–life balance and mental health problems were indicated as common challenges of doctoral students posed by COVID-19 ( Pyhältöa *et al*., 2022). Moreover, the closure of campuses served to exacerbate the more testing elements of the doctoral student experience by intensifying the barriers and psychological risks to students ( Webber *et al*., 2022).

For Dereje, news of military conflict has been “disturbing” and has affected his emotional wellbeing immensely. Badraldeen writes of the time of the COVID-19 pandemic being a time of “massive stress”. During lockdown, he used to stay in his room for long hours and this led him, he believes, into deep depression and anxiety. Also, he adds, the fear of death made him paranoid and he had terrible nightmares. “I felt isolated and depressed, he explains, “and I was in low mode and less productive mode for more than three months.” The “unpredictable political situation in Sudan is another burden”, he adds. This, he explains, is because,

Nobody expects when and how life will return to normal. I can no longer manage to move freely. Meanwhile, the government has no clear vision during such a critical time. All aforementioned situations had huge psycho-social impacts on my life in the mid and long term periods. This has led to anxiety and frustration and I have experienced distress during our working days. I feel powerless due to the low mode of production and performance.

For Tseganesh, news and information about COVID-19 - “the numbers of new cases of COVID-19 infections and deaths that I read in newspapers, and heard on the radio, and watched on the TV” - made her struggle with life and gave her little hope:

I lost focus. I jump here and there without doing anything. I couldn’t get enough sleep. I have talked to my friends for a long time through the phone. But after a while I hated talking to people. I watched movies all day and night. Most of the time I forgot to wear my eyeglasses. Because of that my eyes irritated now and then and I got a headache. I started to hate the couch because I used to sit and sleep there for long time.

Among other things, writes Jean Paul, it was stressful and bothersome to juggle PhD training at home and family duties in difficult moments of the pandemic with lots of uncertainties about whether it would improve. No single day passed, he writes, without asking himself when things would go back to normal:

Sometime I would think if the training will continue to run or suspended. I lived a life of full of uncertainties without knowing what will happen to tomorrow in terms of confinement and restrictions.

### 4. Qualities & Capacities

A final major theme to emerge from our personal experiences of COVID-19 and political and military crisis during the pandemic is that of qualities and capacities this situation has brought out in us, and the different coping strategies we have employed to help us.

## Coping strategies - technology/relationship support

Scholars, such as Sverdlik *et al*. (2022), have underscored how the inability to see family and friends has been among the top challenges facing students during lockdown. In our case, we have been fortunate that one coping strategy we have all employed positively has been seeking support from other people. There have been our friends and family, counsellors, as well as our 5S project team, including fellow PhD students, post docs, our supervisors, and other team members.

Both Tseganesh and Corinna, for example, have relied on their friends’ support to cope with the lockdown and the isolation that came with it. Talking to her friends and sharing how she was feeling motivated Tseganesh to continue working on her PhD project (“I stopped sitting on the couch and tried to deal with the challenge. I started to do my proposal and make it ready for presentation”). It took a while for Corinna to acknowledge how much she was suffering from the isolation and loneliness moving to a new country, England, during the pandemic had imposed on her life. Once she decided to start counselling sessions and share how she was feeling with her supervisors, it became much easier for her to cope as she felt understood and supported. Dereje and Ursin also realised how crucial reaching out and receiving support from supervisors and the 5S team were for their academic journey during these challenging times.

Sharing our situations with and supporting each other as PhD students has helped tremendously. It made us feel connected as a group and thus less alone on our PhD journeys. As we understood and knew about each other’s struggles and challenges, it has made it easier for us to put our individual situations into perspective. Technology (especially social media) has played a major role in keeping us connected to our project and support systems. Keeping up hope and finding a purpose in imposed circumstances has also helped us stay positive.

Even though being under lockdown was not the ideal situation, Badraldeen in Sudan, for instance, made the best out of it by shifting his mindset. He utilised the time spent at home “effectively and efficiently” to read more into and gain a deeper understanding of his PhD topic. Remaining hopeful about the global response to the pandemic and vaccination efforts would quickly ease the circumstances, meanwhile, helped Ursin cope with the situation in Rwanda.

Our experiences have also allowed us to put things into a much bigger picture. Corinna was initially supposed to conduct research in Ethiopia and had prepared for it for over a year, but due to the political conflict, she was not able to go anymore. Although it was initially hard for her to accept, hearing from the Ethiopian, Sudanese, and Rwandan PhD students about their situations made Corinna realise her privileges, especially as a Global North-based student:

Compared to my friends in Ethiopia, Rwanda, and Sudan, just by being Western by birth, I am able to access resources easily, while they often have to struggle to do so. I can rely on stability and social security safety nets, my friends and colleagues cannot. And all of this while already struggling with the unique challenges doing a PhD can pose.

Badraldeen writes, similarly, of a “strong desire to participate in the demonstrations in Khartoum and enthusiasm of being a part of this revolution were much bigger than our own business.” Dereje also recalls how the ongoing conflict in Ethiopia put the challenges he experienced with his PhD into perspective:

It is very sickening to observe all the messes and destructions of the ongoing war on my people. I have spent some sleepless nights and experienced feelings of perplexity because of the conflict. I experienced a feeling of alienation and my study is meaningless if it does not enable me to do something to help the people suffering because of the conflict.

## Conclusion and recommendations

In this article, we have documented our individual and shared experiences of being a PhD student in a period of crisis and our responses at such a time of uncertainty. We described our experiences based in Ethiopia, Rwanda, Sudan, and the UK, through four main themes: New Ways of Working and Its Impact, Change and Delay, Mental Health and Well-Being Impact, and Qualities and Capacities.

The article highlights the challenges involved in conducting research during times of pandemic and in regions susceptible especially to political instability and military conflict. What we hope it illuminates is not only the increasingly well-known psychological and personal impact of undertaking research during the pandemic, but also the added toll that meeting the challenge of the pandemic during times of political crisis and military conflict creates.

The collaborative autoethnographic method has been employed to generate the accounts of the 5S PhD students about their lived experiences of the pandemic and political turmoil. We believe that our article contributes to the development of the emerging collaborative autoethnographic method as a potential technique for documenting and transferring the personal and shared experiences of people.

Our article also provides evidence about the lived experience of being a researcher from/in the Global South amid COVID-19 and the accounts included in this article will serve as additional perspectives in the facets of COVID-19 stories. Our work will hopefully prompt the development of further articles exploring the lived experiences of researchers amid the pandemic and political and military upheavals in different contexts.

We hope that lessons can be learned from our experiences that will serve to minimise the impact of future upheavals caused by similar global and more local events. We also hope that our experiences prompt further work in the area of research and fieldwork safety and care, especially as it relates to ‘home’ researchers. The response of the 5S Foundation to these multiple threats to our doctoral study and personal safety has been flexible, caring, and sympathetic. 5S Foundation management at different levels have been always mindful about new developments and provided pragmatic logistical and pedagogical support to us. While this situation has meant a pause in face-to-face academic activities, the 5S Foundation has worked hard to help us continue at a distance with our research and workshops and training. In particular, we have been provided with access to the internet service through the 5S project’s supply of a Wi-Fi dongle enabling us to remain connected and able to work from home.

The kind of response shown by 5S shows a way forward for responses of this kind in the future. Research institutions and universities, in particular, have an especially crucial role to play. As Grimm *et al*. (2020) write, individuals can only do so much to ensure their own safety, and ultimately academic and research institutions have a duty of care towards their researchers. Research bodies and organisations should therefore, for example, continue pre-arrival to prepare researchers for difficult situations, such as those we have experienced. This institutional support can take a number of different forms ( Grimm *et al*., 2020; Johnson, 2009; Mazurana *et al*., 2013; Mertus, 2009; Morgenbesser & Weiss, 2018; Ogora, 2013).

Lastly, at least one of us has drawn on counselling support during this experience. This article also then underlines the importance of universities and research bodies continuing to provide mental well-being and psychological support, and providing this specifically to those being subjected to the added stress and worry created by a pandemic, political instability, and military conflict.

## Data Availability

Zenodo: Doctoral researchers Covid-19 experiences, collaborative autoethnography dataset https://doi.org/10.5281/zenodo.8327059 ( Bikorimana *et al*., 2023) This project contains the following underlying data: Doctoral researchers covid 19 experiences_Autoethnography_combined.docx Data are available under the terms of the Creative Commons Attribution 4.0 International license (CC-BY 4.0).
