# Supplementary material for: Doctoral research, COVID-19, and political crisis in Ethiopia, Sudan, Rwanda, and the UK: challenges, responses, and recommendations
Source: NIHR Open Res. 2023 Oct 11;3:53. [Version 1] doi: 10.3310/nihropenres.13470.1 (PMC10663879; doi:10.3310/nihropenres.13470.1)
Supplement: Peer review history

# Reviewer report: Reviewer response for version 1

**DOI:** 10.3310/nihropenres.14614.r30641

**Published:** 2023-11-13

**Authors:** Lynette Pretorius

**Affiliations:** 1 School of Curriculum, Teaching and Inclusive Education, Faculty of Education, Monash University, Clayton, Victoria, Australia

I would like to thank the authors for their interesting paper. The topic and the methodology are very closely related to my areas of interest, so I was very interested in this paper. I appreciate the authors' honesty in their reflections, this made this paper a very compelling read.

 The introduction is clearly structured and highlights all the most important aspects of the topic under investigation. It would, however, have been helpful if the introduction also provided a bit of contextual detail about the conflicts/instabilities in Ethiopia, Sudan, and Rwanda for those who may be unfamiliar. This would provide a stronger rationale for why this research is so important. 

 The methodology is well written, providing a clear justification for the methodology choice. However, I do think a bit more detail is needed:

1. In particular, I think you need a bit more of a clear explanation about autoethnography as a methodology and how it links to collaborative autoethnography. I was, for example, surprised not to see Ellis *et al.* referenced, since this is a seminal work in autoethnography. I recently wrote a methodology chapter overviewing the benefits of research in autoethnography and collaborative autoethnography, specifically within COVID-19 times (see chapter reference attached). It may provide you with more references you could use to expand that section to give a stronger justification for why personal stories are so important in this context. 
2. Please specify which prompt questions you used to reflect on your own journeys. This helps to showcase how you applied collaborative autoethnography in a trustworthy manner.
3. You note that collaborative autoethnography has "ethical limitations" - please specify which limitations you mean.
4. I think your use of thematic analysis makes a lot of sense given your topic and the use of collaborative autoethnography as methodology. However, please note that Braun and Clarke ^1^ have updated their methodology and it is now called reflexive thematic analysis (see reference attached as well as their recent 2022 book). This change was made to acknowledge the importance of the reflexivity of the researcher in constructing themes. This is particularly important given your choice of collaborative autoethnography. I suggest you work to provide more detail about how you applied reflexive thematic analysis and how it is related to your collaborative autoethnography methodology. 

The findings and discussion section presents compelling stories of challenges associated with new ways of working, change, mental wellbeing, and capacities. I would, however, have liked to see some more quotes. Collaborative autoethnography as a methodology acknowledges the value of individual lived experiences and the power of stories. As such, using the actual words of the participants a bit more would make the writing more evocative (a key feature of autoethnographic work). For example, the last quote of "coping strategies" is really powerful. More of these types of quotes would greatly benefit your paper.

 Thank you for this powerful piece.

Is the work clearly and accurately presented and does it cite the current literature?

Yes

If applicable, is the statistical analysis and its interpretation appropriate?

Not applicable

Are all the source data underlying the results available to ensure full reproducibility?

Yes

Is the study design appropriate and is the work technically sound?

Yes

Are the conclusions drawn adequately supported by the results?

Yes

Are sufficient details of methods and analysis provided to allow replication by others?

Partly

Reviewer Expertise:

Doctoral Education, Academic Identity, Student Wellbeing, Reflection, Autoethnography, Doctoral Writing Groups, Pastoral Care, Experiential Learning, Academic Writing, Academic Language, Academic Literacy, Academic Integrity, Belonging, Collaborative Learning, Research Skills, Transferable Skills, Qualitative Methods, Mixed Methods, Education for Social Justice

I confirm that I have read this submission and believe that I have an appropriate level of expertise to confirm that it is of an acceptable scientific standard.

## References

[1] : Reflecting on reflexive thematic analysis. *Qualitative Research in Sport, Exercise and Health* .2019;11(4) : 10.1080/2159676X.2019.1628806 589-597 10.1080/2159676X.2019.1628806

# Reviewer report: Reviewer response for version 1

**DOI:** 10.3310/nihropenres.14614.r30572

**Published:** 2023-10-27

**Authors:** Ryan Thomas Williams

**Affiliations:** 1 Teesside University, Middlesbrough, England, UK

The introduction emphasises the inherent challenges of conducting doctoral research, which helps establish context. The statements are well-supported with citations, adding credibility to the claims made. Connecting the doctoral journey with the impact of the COVID-19 pandemic and political unrest makes the study timely and relevant.

 The methods are well-detailed and provide a comprehensive understanding of the approach taken for the research.

 The specific timeline ("we started planning for our collaborative research and writing our experience in September 2021, and the manuscript had been fully written in June, 2022”) is excellent as it provides a clear timeframe for your research.

 It would be beneficial to go into more depth regarding how the thematic analysis was conducted, especially given its importance in the study. For instance, how were discrepancies in initial codes resolved? How were overarching themes validated?

 Your results section offers a detailed account of the challenges faced during the pandemic and political unrest. It successfully captures the nuanced experiences from different contexts, providing rich insights into the lived realities of researchers in these regions. 

 The article excels in giving voices to individual experiences. These personalised stories enhance the depth of the topic and make it relatable for the readers.

 While the challenges are critical, hearing about unexpected positive outcomes or silver linings that emerged from these experiences might be refreshing. Badraldeen’s account offers a glimpse into this, but more could balance the narratives.

 The conclusion is coherent and clearly states the research’s key findings and implications. This article addresses critical challenges PhD students face, especially from/in the Global South, during a crisis.

Is the work clearly and accurately presented and does it cite the current literature?

Yes

If applicable, is the statistical analysis and its interpretation appropriate?

Not applicable

Are all the source data underlying the results available to ensure full reproducibility?

Partly

Is the study design appropriate and is the work technically sound?

Yes

Are the conclusions drawn adequately supported by the results?

Yes

Are sufficient details of methods and analysis provided to allow replication by others?

Yes

Reviewer Expertise:

Education technology, internet ethics, reflective practice, reflexivity, mixed methods

I confirm that I have read this submission and believe that I have an appropriate level of expertise to confirm that it is of an acceptable scientific standard.
